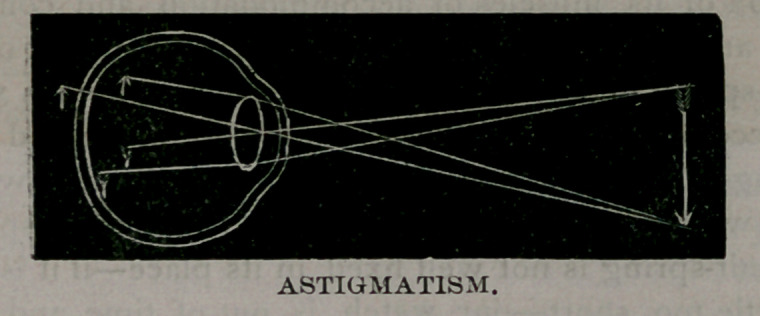# The Relation of Errors of Refraction and Accommodation to Defective Sight, and to Some Nervous Disturbances

**Published:** 1884-03-20

**Authors:** A. G. Hobbs

**Affiliations:** Prof. of Eye, Ear and Throat Diseases and Lecturer on Dermatology, Southern Medical College, Atlanta, Georgia


### Southern Medical Record:

EDITORS:

THOMAS S. POWELL, M.D. R. C. WORD, M.D.

*R. C. WORD, M.D., Managing Editor.*

☞All Communications and Letters on Business connected with the RECORD must be addressed to the Managing Editor.

VOL. XIV. ATLANTA, GA., MARCH 20, 1884. No. 3.

### ORIGINAL AND SELECTED ARTICLES.

### THE RELATION OF ERRORS OF REFRACTION AND ACCOMMODATION TO DEFECTIVE SIGHT, AND TO SOME NERVOUS DISTURBANCES.

BY A. G. HOBBS, M. D.,

**Prof. of Eye, Ear and Throat Diseases and Lecturer on Dermatology, Southern Medical College, Atlanta, Georgia.**

Should a person wear glasses when glasses enable him to see more plainly and are a source of comfort to him, is no longer a question, any more than it is a question that he should wear a brace in Potts' disease to support the weight of the spinal column from bearing down upon the diseased bone.

To wear glasses when glasses are beneficial is no longer considered "affected" or "foppish," any more than to use a cane when a cane assists a lame leg.

The old idea that one should postpone the use of glasses as long as possible, even at the sacrifice of comfort and good vision, and possibly of healthy lids or of perfect nervous functions, is is long since exploded. The old adage was: "Refrain from glasses just as long as possible, because if you begin them you *cannot* leave them off." If, in the above reason, *will not* be substituted for *cannot*, it will come nearer the truth, since he who needs them and uses them *will not* give them up because of the comfort and assistance they render him, and not because he sees worse without them than he did before he began their use.

Anomalies of refraction and accommodation enter into the etiology of defective vision and many nervous derangements to a far greater extent than many practitioners conceive or will admit. It is unfortunate that the impression should prevail among so many that this subject is one of unusual difficulty. A moderate amount of application will render any physician able to, at least, recognize the difference between these and other causes of disturbed vision.

By making reference to some of the laws of light, the subject can be made simpler. For example: "Rays of light passing from a rarer to a denser medium are refracted towards the perpendicular;" then, in passing from the air into the refractive media of the eye, the rays of light are refracted towards the perpendicular and, hence, meet and focus on the retina in an emmetropic (normal) eye, Anterior to the retina in a myopic (near-sighted) eye and posterior to the retina in a hyperopic (over-sighted) eye, provided the accommodation is in a quiescent state and the rays of light are parallel, *i. e.*, come from a distance.

Then, in the emmetropic eye (the ideally correct eye) while in a state of rest, *i. e.*, while accommodation is not exercised, rays of light proceeding from a distant object are brought to a focus by the refractive mediæ (cornæ, aqueous, lens and vitreous) upon the percipient layer of the retina. Practically, rays of light proceeding from an object twenty feet or more distant are parallel; within that distance, divergence begins and increases as the object approaches; and as the rays become more and more divergent—as the object approaches—they are focussed farther and farther behind the retina of the normal eye, if it remain quiescent; but if the eye accommodates, *i. e.*, if the lens becomes more convex, the increased divergent rays are neutralized and the focus falls again upon the retina. Hence, we see, accommodation is exercised—an effort is kept up—even in the most perfect eye; for example, in reading.

This explains how the most natural eye, if kept reading or at close work for a long time, will feel tired and ache, and perhaps the letters will appear blurred. The muscles of accommodation have become tired, just as any other muscle will tire if overworked. Rest is the natural remedy; but, if this be out of the question, the next best thing is to place a pair of weak convex glasses (the number suited to each case) in front of the eye to lessen the necessity of so much accommodation. This power, called accommodation, is brought about by the contraction of the ciliary muscle, which, by its contraction, relaxes the suspensory ligament and permits the crystalline lens, by its own elasticity, to become more convex and, hence, increase its focal power. Accommodation becomes stronger and stronger as the object approaches nearer and nearer the eye. Up to a certain point vision is clearer, because the retinal image is large; but when that point, called the "near point," is reached, vision is no longer clear, because accommodation can no longer overcome the extreme divergence, and the focus is thrown behind the retina.

In young persons and in myopes this "near point" is very much nearer than in older persons, in whom the lens has become naturally harder. For example: a man of 45 or 50 holds his newspaper at arm's length, that the rays of light leaving the page may enter the eye as nearly parallel as possible. To demonstrate, bring this page gradually nearer your eyes and the letters will blurr when it has reached to within 3 to 6 inches; now introduce before your eye a card with a pin-hole in it and the "near point" will approach nearer, because the small opening through which the letters are seen excludes the external or divergent rays and admits only the central ones, or those which are nearly parallel.

We have stated that in early life the lens is soft and this effort of the ciliary muscle which produces accommodation is most easily accomplished and maintained; but when the age of 45 or 50 is reached the lens becomes so hard that its elasticity is diminished; hence, accommodation is diminished and the "near point" recedes to eighteen inches or two feet. This condition is called presbyopia (old sight). It is purely a physiological change and should not be regarded with anxiety. It should be corrected by properly adjusted convex glasses for reading, that the page may be held closer to give a larger retinal image. A convex lens is placed in front of the eye to add to the power of the hardened crystalline lens.

In order that the effort of accommodation may be kept up for the greatest length of time without tiring the normal eye, two things are necessary: a good light, adjusted so that its rays may fall well upon the page, for instance, in reading, and the page should be held as far from the eye as perfect distinctness will admit. For example, when the "near point" is a distance of eight inches, prolonged adjustment cannot be sustained unless the page be held at 16 or 20 inches.

EMMETROPIA.

An emmetropic eye is the one, the refractive mediæ of which are able without the aid of accommodation to focus parallel rays upon the retina.

MYOPIA.

In a myopic or near-sighted eye, parallel rays of light are focussed before reaching the retina and, therefore, the eye is longer than the focal distance of its refractive media.

HYPEROPIA.

In hyperopia or hypermetropia (over-sight), the focus falls beyond the retina and, consequently, the eye is shorter than its focal distance."

A person with normal eyes who looks through strong convex or concave glasses is beset with the same difficulties as the individual who has uncorrected hyperopia or myopia.

The hyperope is compelled to overtax his muscles of accommodation while the muscles of convergence (the internal recti) are only normally acting, and the myopic person strains the muscles of convergence while the ciliary muscles are almost at rest.

It will be seen that a muscular effort of the internal recti is always necessary in addition to the effort of accommodation; because, in bringing an object near the eyes, it is necessary that the eyes should become more convergent in order that the retinal image in each eye should fall exactly upon the macula lutea, by which means alone is single vision from two eyes obtained. Motor branches of the same nerve supply the internal recti and ciliary muscles; so that, although they act independently, they should act together; hence, when, from any cause, they act unequally, a disturbance of vision or pain is the result. This can be demonstrated by any one with a normal eye. Such a person may read with the page at a proper distance from the eye or look at a distant landscape an almost indefinite length of time without experiencing any inconvenience, but if he should attempt to read with strong convex glasses, or to look in the distance with strong concave glasses, the harmony in the action of the recti muscles and the ciliary muscles is disturbed and a discomfort or a pain in the eyes is felt.

ASTIGMATISM.

Astigmatism is a refractive error productive of more disturbance, and is more difficult to correct, than either myopia, hyperopia or presbyopia. It is also liable to produce more nervous manifestations. In this condition either the cornea or the lens, generally the former, is irregular, and, as a consequence, an imperfect or distorted image is produced upon the retina. One may obtain an idea of this condition by looking through an imperfect window-glass at the distorted objects. One meridian may focus the rays of light that pass through it anterior to the retina, and another meridian posterior to the retina. One meridian may fall upon the retina, and another either anterior or posterior to it, or all meridians may fall anterior, or all posterior to the retina. This error (regular astigmatism), then, manifestly, may complicate either emmetropia, hyperopia or myopia. For this reason we have simple astigmatism, either hyperopic or myopic, and compound astigmatism, both hyperopic and myopic; and there may be mixed astigmatism, in which either hyperopia or myopia may predominate.

All of these well known facts have been mentioned only that the reader may more clearly understand the views of some late authors on the relation which exists between some errors of refraction and some so-called functional nervous diseases.

That blepharitis is frequently caused and kept up by errors of refraction, especially hyperopia, was demonstrated some years ago by Dr. Roosa, of New York, and has since been many times confirmed; indeed, it is often impossible to cure this disease without correcting the refractive error.

Any one who has observed the annoyance produced by an uncorrected hyperopia or astigmatism can readily understand how they may cause serious nervous disturbances.

Dr. J. E. Harper says: "As a general rule, certain forms of visual defects and functional nervous diseases are in relation to each other as cause and effect."

"That neuralgia, vertigo, the common forms of headache, epilepsy, chorea, hysteria and certain forms of insanity may have their origin in defects in the shape of the eye, requiring an unnatural exertion of its muscles of accommodation and convergence." He gives an apt simile: "We may compare the heart of a man to the main-spring of a watch, while the delicate nerves which control the accommodation of the eye may be compared to the fine hair-spring of the time-keeper. It matters little how often we wind the watch, and thus supply tension to the main-spring, if the delicate hair-spring is not well fixed in its place—if it is a little too long, a little too short—our watch is out of time and cannot be made to run true; but regulate the tension of this extremely delicate spring and the movement of the watch becomes perfect and reliable. So, the heart may supply the force necessary for the perfect working of the human machine; but if the tension upon these delicate nerves is not well regulated there may be such nervous disturbance as seriously to impair the comfort and usefulness of the individual. There may be a broken cog or an imperfect wheel in the time-keeper which, in spite of main-spring or regulator, will sooner or later stop the watch; and in the human machine there may be germs, organic lesions, which will disturb or destroy its workings; but, excepting these, and other things being equal, the boy or girl with the best heart and the best eye is the most completely armed for the battle of life."

When errors of refraction occur in children, they are more often overlooked than in adults, because such troubles are popularly believed to be peculiar to adult life. If the cause is not apparent, the child is accused of maligning or of inattention. When such troubles occur in children they should be immediately examined, the cause found and corrected; if not, application to books will intensify the trouble and it will grow worse as the child grows older.

Most all errors of refraction can be easily corrected if no other defect exists. We are often asked: "Doctor, can you not give me good sight in some other way than fitting glasses to my eyes?" A man with one leg might as well ask: "Can I not have my leg restored without having to use an artificial one?" Art made a long stride when she furnished the almost perfect artificial leg; but more good has been wrought since we have been enabled to adjust glasses to almost any error of refraction in such a manner that vision is, in many cases, perfectly restored.

"Formerly, when a person reached 45 or 50 years of age, and found that he was forced to trombone his newspaper in order to find the least uncomfortable distance at which to hold it, he sought the nearest jeweler, bought a pair of spectacles which seemed nearest right, but which were often most completely wrong, concluded that he was growing old and losing his powers, and so, with as good grace as possible, submitted to the greatest cross and humiliation of his life."

Physicians should endeavor to correct the erroneous popular impression that exists in regard to spectacles. Statistics have time and again proven that nearly one-fourth of the children in our schools have some anomalous refraction, and, since our present system of education has brought about this condition, it only remains for us to try to ameliorate the condition as best we can. Properly fitting glasses will not only often check the progress of a myopia or hyperopia in a child, but will often lessen the refractive error, so that after a few months or years they may be left off entirely.

Dr. G. F. Stevens, in an article upon this subject, says: "The wearing of glasses for the correction of errors of refraction is neither an indication of age or of foppery, but a sensible acceptance of the great benefits which science confers. No longer do those who are well informed in respect to such troubles seek a shop where glasses are kept on sale; for one might about as well, if he desired a photograph taken, request his neighbor to sit for him or search through the show-cases of the photographer for a good picture of himself. He must sit for his own photograph and he must sit for his own glasses, and often the highest scientific knowledge of the surgeon and the most consummate skill of the artisan are brought into requisition for the correction of a refractive error of the very existence of which the patient is scarcely aware."

Hitherto this knowledge of the surgeon and skill of the worker in glass have been, almost exclusively, of service only when the patient complains of visual troubles; henceforth, I am convinced that these visual troubles will be sought for in order to afford relief, for the protection against many nervous affections.

---

A VOTE ON THE CODE QUESTION in the State of New York, according to the Medical News, shows that of 2943 physicians, 2256 are in favor of the old code, and only 687 in favor of the innovation.

## Figures and Tables

**Figure f1:**
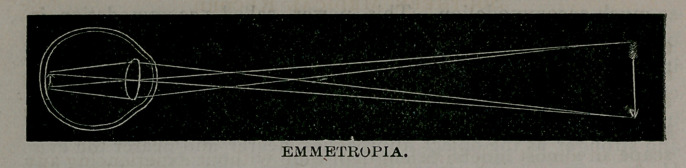


**Figure f2:**
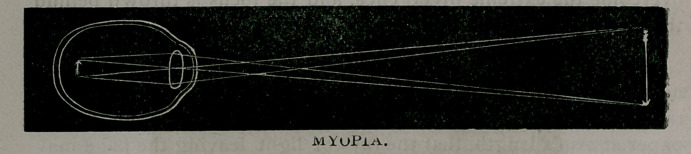


**Figure f3:**
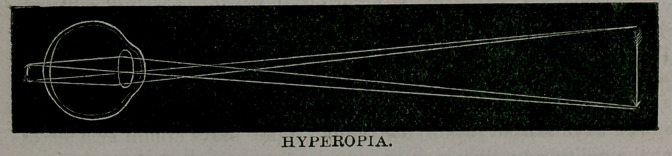


**Figure f4:**